# Prenatal Human Milk Oligosaccharides (HMOs) in the Context of BMI, Gestational Weight Gain, and Lipid Profile—An Association Study in Pregnant Women with Overweight or Obesity

**DOI:** 10.1002/mnfr.202300533

**Published:** 2023-12-12

**Authors:** Lukas Schönbacher, Carmen Treichler, Waltraud Brandl, Harald C. Köfeler, Herbert Fluhr, Evelyn Jantscher‐Krenn, Mireille N. M. van Poppel

**Affiliations:** ^1^ Department of Obstetrics and Gynecology Medical University of Graz Auenbruggerplatz 14 Graz 8036 Austria; ^2^ BioTechMed‐Graz Mozartgasse 12/II Graz 8010 Austria; ^3^ Center for Medical Research Medical University of Graz Stiftingtalstraße 24 Graz 8010 Austria; ^4^ Institute of Human Movement Science Sport and Health University of Graz Mozartgasse 14/I Graz 8010 Austria

**Keywords:** 2’‐Fucosyllactose (2’FL), FUT2, human milk oligosaccharides (HMOs), obesity, pregnancy

## Abstract

**Background:**

Human milk oligosaccharides (HMOs) are bioactive glycans first detected in human milk. Their presence in maternal blood during pregnancy suggests systemic functions. Dynamics and associations of the most abundant prenatal HMOs in relation to maternal BMI and serum lipids in a cohort of 87 pregnant women with either overweight or obesity are studied.

**Methods:**

Serum HMOs (2’FL, 3’SL, 3’SLN, LDFT), serum lipids (total cholesterol, HDL, LDL, triglycerides), and BMI are measured at 15, 24, and 32 weeks of gestation.

**Results:**

2’FL and LDFT are negatively correlated to pre‐pregnancy BMI and increase significantly slower between 15 and 24 weeks in highly obese women. Women without detectable increase of serum 2’FL (non‐secretors) show a less pronounced gestational weight gain and lower BMI in the third trimester as compared to women phenotype as secretors. Higher early‐pregnancy 2’FL is associated with high HDL and low triglycerides in pregnancy. On the other hand, higher 3’SL at 15 weeks is associated with higher triglycerides, LDL, and total cholesterol.

**Conclusions:**

Higher early‐pregnancy 2’FL is associated with a cardioprotective lipid profile, whereas higher 3’SL is associated with an atherogenic lipid profile. Serum trajectories of 2’FL and LDFT in obese women suggest an obesity mediated delay of α‐1,2‐fucosylation.

## Graphical abstract

This study investigates human milk oligosaccharides (HMOs) in maternal serum during pregnancy in relation to maternal BMI, gestational weight gain, and serum lipids. 2'‐Fucosyllactose (2’FL) is associated with a beneficial lipid profile (high HDL, low triglycerides), and is reduced in highly obese women, whereas 3'‐Sialyllactose (3’SL) is associated with an atherogenic lipid profile (high triglycerides, LDL, and total cholesterol).

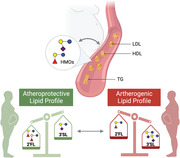

## 1 Introduction

Human milk oligosaccharides (HMOs) are a group of more than 150 structurally distinct, bioactive, and human‐specific glycans featured in human milk.^[^ ^1^, ^2^ ^]^ HMOs have been postulated to account for many benefits in the breast‐fed infant, including prebiotic,^[^ ^3^ ^]^ anti‐infective,^[^ ^4^, ^5^ ^]^ and immune‐modulating functions.^[^ ^6^ ^]^ They have been shown to influence proliferation and differentiation of epithelial cells^[^ ^7^ ^]^ and mucosal immune cells.^[^ ^8^, ^9^ ^]^ Since HMOs can be absorbed in the gut and enter the systemic circulation of breast‐fed infants,^[^ ^10^, ^11^ ^]^ their activity is not restricted to the gut. In vivo and in vitro studies revealed HMOs as signaling molecules in both a pro‐ and anti‐inflammatory manner^[^ ^12^, ^13^, ^14^, ^15^ ^]^ and also suggested immunomodulating and metabolic functions,^[^ ^16^ ^]^ opening doors to a wide field of yet sparsely investigated systemic functions.

The presence of HMOs is not limited to human milk and the lactation period. HMOs already emerge early in pregnancy in the maternal systemic circulation, and concentrations increase with gestational age. Previous studies characterized HMOs in maternal serum and urine^[^ ^17^, ^18^, ^19^ ^]^ during the course of pregnancy, as well as in umbilical cord blood and amniotic fluid,^[^ ^20^, ^21^ ^]^ strongly suggesting an extensive exposure of the mother‐placenta‐fetus unit to HMOs. However, the biological roles of HMOs in pregnancy remain largely elusive. Addressing potential involvements in metabolic adaptation processes during pregnancy, we showed in a previous study that serum concentrations of the sialylated HMOs 3’SL and 3’SLN were associated with higher fasting glucose levels. In the same cohort, 3’SL early in pregnancy was predictive for the later diagnosis of gestational diabetes (GDM).^[^ ^18^ ^]^ Knowing of associations to glucose metabolism during pregnancy encourages to further investigate potential associations of serum HMOs to obesity, body weight gain, and lipid metabolism during pregnancy.

Overweight and obesity are known to increase the risk for dyslipidemia and alter trajectories of blood lipids during gestation.^[^ ^22^ ^]^ Physiological pregnancy is characterized by rising levels of total plasma cholesterol, low density lipoproteins (LDL), high density lipoproteins (HDL), and triglycerides.^[^ ^23^ ^]^ Dyslipidemia, i.e., increased levels of total cholesterol, LDL, and triglycerides, is associated with pregnancy complications such as preeclampsia^[^ ^24^ ^]^ and GDM.^[^ ^25^ ^]^ High triglycerides and low HDL are associated with increased birth weight and higher risk for macrosomia, especially in women with overweight or obesity.^[^ ^26^ ^]^ Besides pregnancy, dyslipidemia is a well‐known risk factor for atherosclerotic cardiovascular diseases.^[^ ^27^ ^]^

Studies in rodent models indicate that 2’FL, one of the most abundant serum HMOs during pregnancy, can influence lipid metabolism and weight development. Oral supplementation with 2’FL has been shown to be protective against liver steatosis in obese mice by reducing microvesicular steatosis, activating lipid catabolism, and deactivating lipogenesis.^[^ ^28^ ^]^ Another study in mice showed that oral supplementation of 2’FL led to improvement of the hyperphagic response to a high fat diet.^[^ ^29^ ^]^

2’FL is an α‐1,2‐fucosylated HMO whose abundance is strongly influenced by the genetic background. Polymorphisms in the *Secretor* gene (Se) encoding for α‐1,2‐fucosyltransferase 2 (FUT2) determine an individual's secretor phenotype. Women with a negative secretor status (non‐secretors) are lacking FUT2 activity and thus, α‐1,2‐fucosylated HMOs, especially 2′FL, which is the predominant serum HMO in secretor positive women (secretors).^[^ ^30^ ^]^ Since most human populations are characterized by a considerable percentage of secretor negative women (who can be seen as natural knockouts for FUT2), there is the opportunity to study differences between the two groups: women lacking 2’FL (secretor negative women) and those with overall high amounts of 2’FL (secretor positive women). Hence, potential impact of systemic 2’FL on lipid metabolism and weight gain during pregnancy can be examined by comparing secretors and non‐secretors.

Besides the question, how HMOs (e.g., 2’FL) influence body weight gain and lipid metabolism during pregnancy, it is of interest, whether (pre‐pregnancy) BMI has an effect on the composition of serum HMOs. Recent studies suggest that maternal obesity is associated with concentrations of HMOs in human milk.^[^ ^31^, ^32^, ^33^ ^]^ While results are inconsistent as to the direction of the effect on individual HMOs in milk, even less is known about the influence of maternal pre‐pregnancy BMI on serum HMOs during pregnancy. In a pilot study in healthy, mainly normal weight pregnant women, we recently reported a negative association of serum 2’FL concentrations with pre‐pregnancy BMI and subcutaneous adipose tissue (SAT).^[^ ^17^ ^]^ However, it is not clear whether this also holds true for a population of pregnant women with overweight or obesity that has an increased risk for metabolic derangements such as gestational diabetes and dyslipidemia.

To answer these questions, we quantified the four most common serum HMOs and serum lipids during the course of pregnancy in a well phenotyped cohort of 87 pregnant women with either overweight or obesity. We further aimed to a) investigate the influence of serum 2’FL on body weight gain by comparing secretors and non‐secretors, b) evaluate an impact of pre‐pregnancy BMI on HMO concentrations, and c) assess associations between HMOs and serum lipids.

## 2 Results

**Figure 1:**
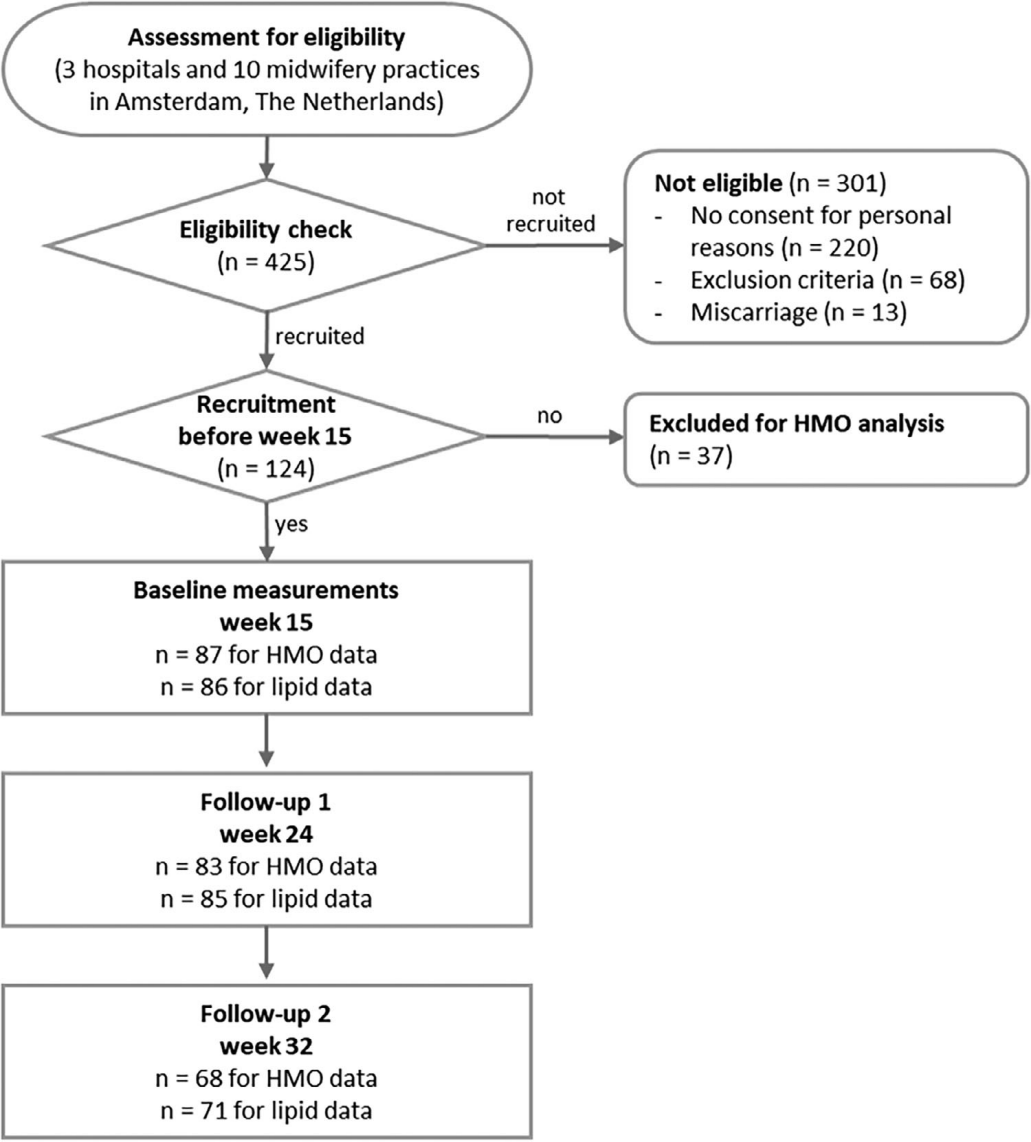
Flowchart of recruitment and follow‐up of study participants.

Data of 87 participants were available for the analyses (**Figure** **1**). Baseline characteristics of the participants are presented in **Table** **1**. Women were on average 30.2 (±5.0) years of age, 40% of Caucasian origin, and 37% primiparous. All of them were either overweight or obese (BMI > 25 kg m^−^^2^) already before pregnancy, representing a BMI range from 25 to 54 kg m^−^^2^ with an average BMI of 33.3 (±4.9) kg m^−^^2^. 17% of all participants were considered secretor negative (15 of 87 women); their basic characteristics showed no differences to secretor positive women.

**Table 1:** Baseline characteristics of the total sample and per secretor status.

	Total *N* = 87	Secretor positive *N* = 72	Secretor negative *N* = 15	*p*
Age, years, *n* = 87	30.2 ± 5.0	30.4 ± 5.2	29.7 ± 4.2	0.63
BMI pre‐pregnancy, kg m^−2^ *n* = 87	33.3 ± 4.9	33.2 ± 4.6	33.8 ± 6.2	0.66
Primiparous *n* = 81	30 (37%)	20 (35%)	5 (42%)	0.67
Married *n* = 81	37 (46%)	27 (41%)	10 (67%)	0.07
Education *n* = 80				0.34^a^ ^)^
Low	27 (34%)	21 (32%)	6 (43%)	
Middle	31 (39%)	28 (42%)	3 (21%)	
High	22 (28%)	17 (26%)	5 (36%)	
Caucasian *n* = 81	35 (40%)	30 (46%)	5 (33%)	0.39
Smoking *n* = 81	6 (7%)	5 (7%)	1 (7%)	0.13

Means ± SD or *N* (%); BMI, body mass index; GWG, gestational weight gain; student *t*‐test.

^a)^ Chi‐square test.

### 2.1 HMOs and Most Lipids Increase throughout Pregnancy

**Figure 2:**
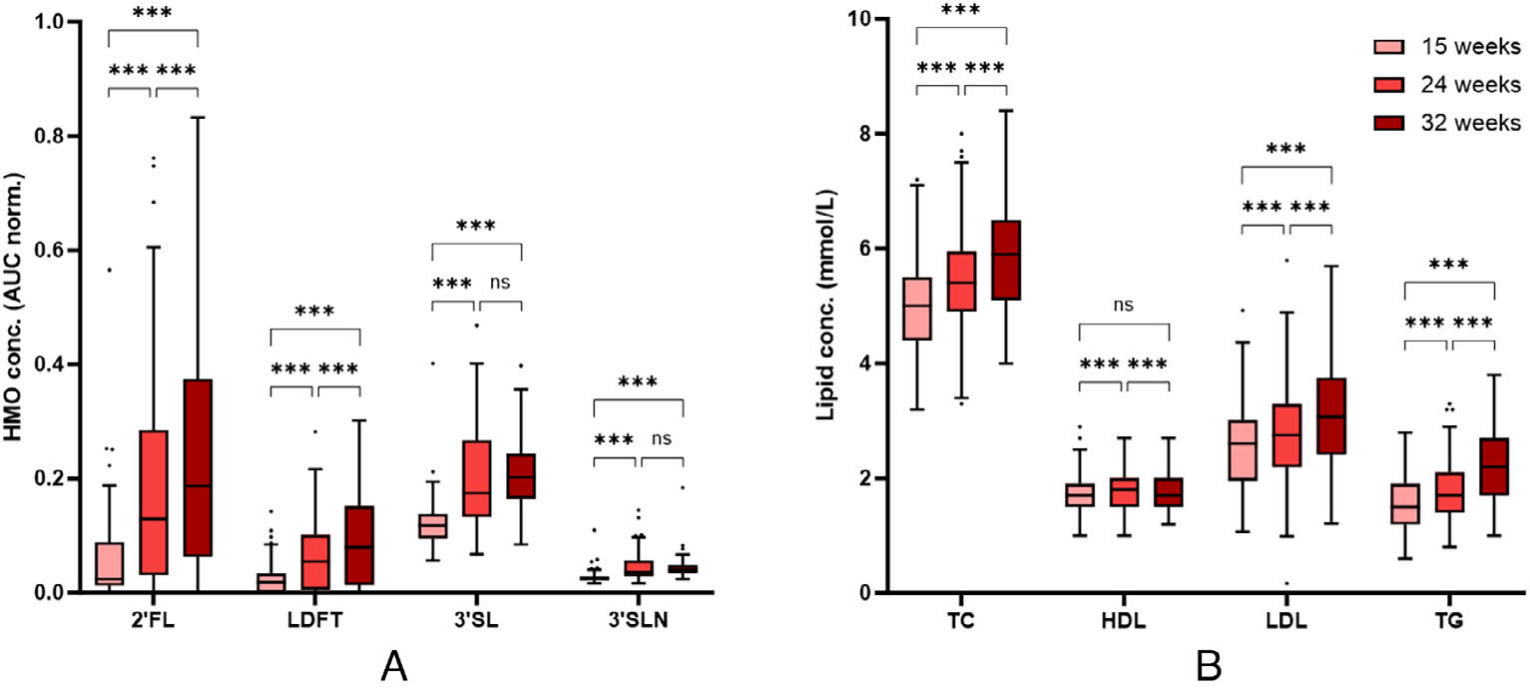
Concentrations of serum HMOs **(A)** and lipids **(B)** over the course of pregnancy. Box‐and‐whisker‐plots (Tukey) show increasing concentrations of all four HMOs. Total cholesterol (TC), LDL, and triglycerides (TG) increase with each study visit, HDL shows highest concentrations at 24 weeks. (ns, not significant; *** *p* < 0.001).

**Figure** **2** shows concentrations of HMOs and serum lipids. As previously reported, all four investigated HMOs significantly increased in concentration throughout pregnancy from week 15 to 32 (also see Table S1, Supporting Information) (Friedman ANOVA *p* < 0.001).^[^ ^18^ ^]^ Per definition, 2’FL and LDFT were significantly higher in secretor positive women, but sialylated HMOs 3’SL and 3’SLN showed no difference between secretor positive and negative women (*p* > 0.05, data not shown).

Total cholesterol, LDL, and triglyceride concentrations significantly increased from 15 to 32 weeks of pregnancy. HDL concentrations were highest at 24 weeks, but at 32 weeks returned to a concentration similar to the concentration at 15 weeks. Lipid concentrations were not different between secretor positive and negative women at 15 weeks or 24 weeks. At 32 weeks, HDL was higher in secretor positive women compared to secretor negative women (1.80 ± 0.38 mmol L^−1^ vs 1.51 ± 0.24 mmol L^−1^, *p* = 0.01).

In comparison to reference intervals for serum lipids of pregnant women, as recently published by Lu et al.,^[^ ^34^ ^]^ a conspicuously large proportion of our cohort exceeded the upper reference limit (URL) at week 15, indicating an increased prevalence of dyslipidemia: 14.1% exceeded the URL for total cholesterol (>5.82 mmol L^−1^), 12.0% for LDL (>3.61 mmol L^−1^), and 12.0% for triglycerides (>2.2 mmol L^−1^). None of our participants undercut the lower reference limit for HDL (<1.0 mmol L^−1^).

### 2.2 Secretor Status Influences Trajectories of Gestational Weight Gain

**Table** **2** shows BMI and gestational weight gain (GWG) for the total study sample as well as separately for secretor positive and negative women. There was no difference in early‐ and pre‐pregnancy BMI between secretors and non‐secretors, but at week 32 secretor negative women showed a significant lower BMI.

**Table 2:** BMI and GWG at different time points (total sample and per secretor status).

	Total	Secretor positive	Secretor negative	*P*
BMI pre‐pregnancy, kg m^−2^ *n* = 87	33.3 ± 4.9	33.2 ± 4.6	33.8 ± 6.2	0.66
BMI at 15 weeks, kg m^−2^ *n* = 87	34.6 ± 4.9	34.5 ± 4.6	34.9 ± 6.1	0.82
BMI at 24 weeks, kg m^−2^ *n* = 82	35.3 ± 4.6	35.6 ± 6.2	33.8 ± 4.3	0.19
BMI at 32 weeks, kg m^−2^ *n* = 71	36.4 ± 4.7	36.8 ± 6.2	33.8 ± 3.1	**0.048**
GWG, kg *n* = 71	5.8 ± 4.0	6.1 ± 3.8	4.0 ± 5.0	0.14
GWG per week, 15–24 weeks, kg wk^−1^; *n* = 82	0.31 ± 0.42	0.36 ± 0.36	0.03 ± 0.59	**0.007**
GWG per week, 24–32 weeks, kg wk^−1^, *n* = 71	0.37 ± 0.24	0.40 ± 0.24	0.26 ± 0.27	0.097
Excessive GWG at 24 weeks; *n* = 82	53 (64.6%)	48 (64.9%)	5 (36.7%)	**0.041** ^a^ ^)^
Excessive GWG at 32 weeks; *n* = 71	47 (66.2%)	40 (66.7%)	7 (63.6%)	0.85^a^ ^)^

Mean ± SD or *N* (%); BMI, body mass index; GWG, gestational weight gain; student *t*‐test.

^a)^ Chi‐square test.

Women who were non‐secretors had a significantly reduced weekly weight gain between week 15 and 24 compared to secretors. The same trend was observed between week 24 and 32 but did not reach significance. Analogously, the rate of women with excessive GWG (defined as exceeding the IOM recommendations for women with overweight or obesity) was significantly lower within non‐secretors compared to secretors at week 24.

**Figure 3:**
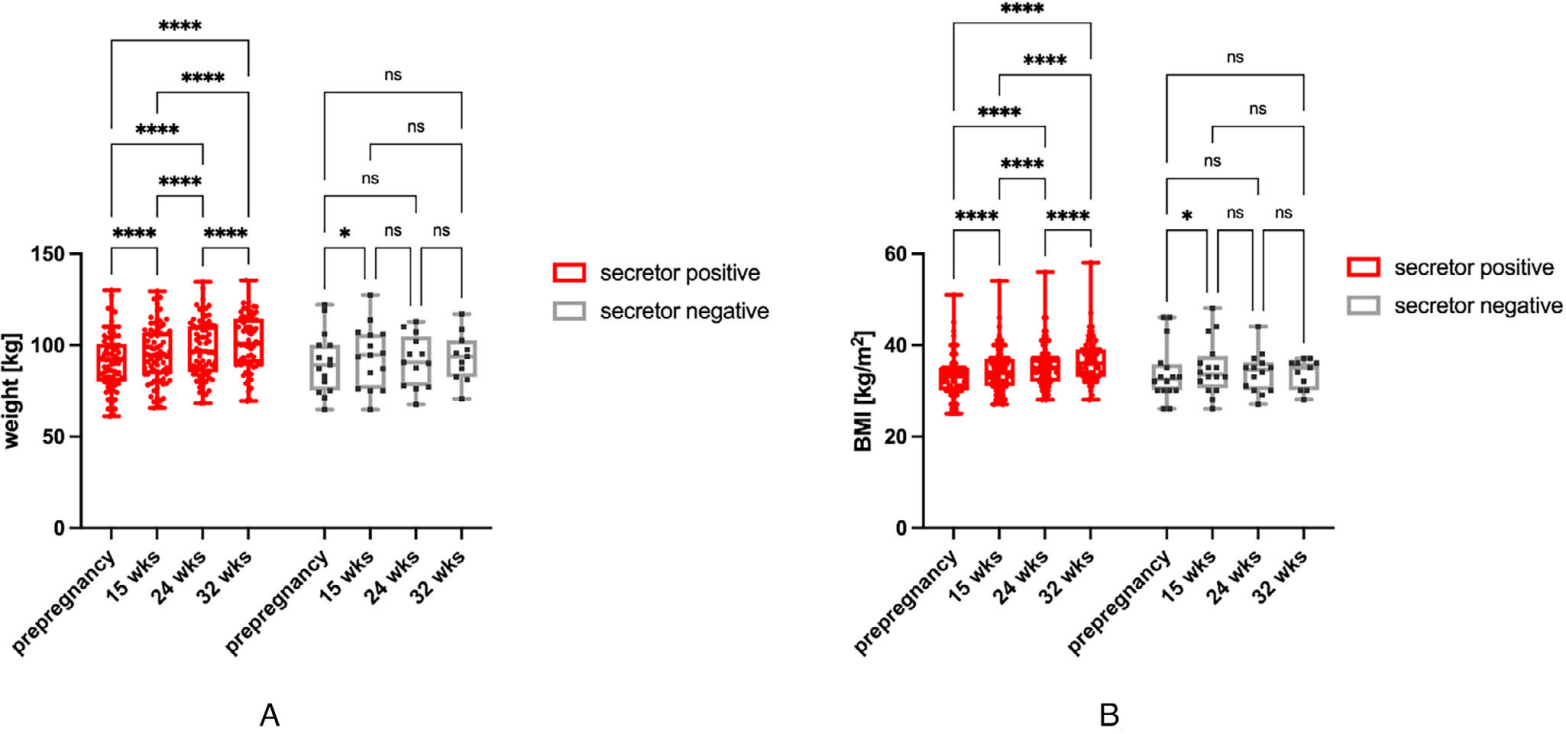
Body weight and BMI over the course of pregnancy stratified by secretor status. Box‐and‐whisker‐plots show body weight **(A)** and BMI **(B)** separately for secretor positive and secretor negative women at different time points in pregnancy. There is a steady increase in weight and BMI in secretor positive women, but not in secretor negative women (ns, not significant; *p < 0.05; **** *p* < 0.0001).

In the secretor positive group, the mean BMI steadily increased over the course of pregnancy. However, within the secretor negative group, the mean BMI was highest at 15 weeks of gestation, and later returned to a level comparable to pre‐pregnancy BMI with no further increases (**Figure** **3**, Table 2).

### 2.3 Women with BMI ≥35 kg m^−^^2^ Show a Delayed Increase in Fucosylated HMOs over Gestation

We next investigated whether pre‐pregnancy BMI was associated with HMO concentrations in women with overweight or obesity. **Table** **3** shows Spearman rank correlations between pre‐pregnancy BMI and HMOs at different time points. Due to the lacking capability of producing 2’FL and LDFT, secretor‐negative women were excluded from correlation analyses of these HMOs. A significant negative correlation was found between pre‐pregnancy BMI and 2’FL at week 24. LDFT showed even stronger significant negative correlations with pre‐pregnancy BMI at week 24 and 32. No correlations were found for pre‐pregnancy BMI with 3’SL and 3’SLN.

**Table 3:** Spearman correlations between pre‐pregnancy BMI and HMOs at different time points.

	Week 15	Week 24	Week 32
		2’FL (secretor positive only)	
Spearman coefficient	−0.151	**−0.286**	−0.187
*p*	0.204	**0.015**	0.153
*N*	72	**71**	60
		LDFT (secretor positive only)	
Spearman coefficient	−0.176	**−0.335**	**−0.300**
*p*	0.139	**0.004**	**0.020**
*N*	72	**71**	**60**
		3’SL (total cohort)	
Spearman coefficient	0.088	−0.051	−0.017
*p*	0.418	0.639	0.888
*N*	87	86	72
		3’SLN (total cohort)	
Spearman coefficient	0.039	−0.016	0.141
*p*	0.721	0.884	0.238
*N*	87	86	72

Bold font indicates statistically significant correlations.

**Figure 4:**
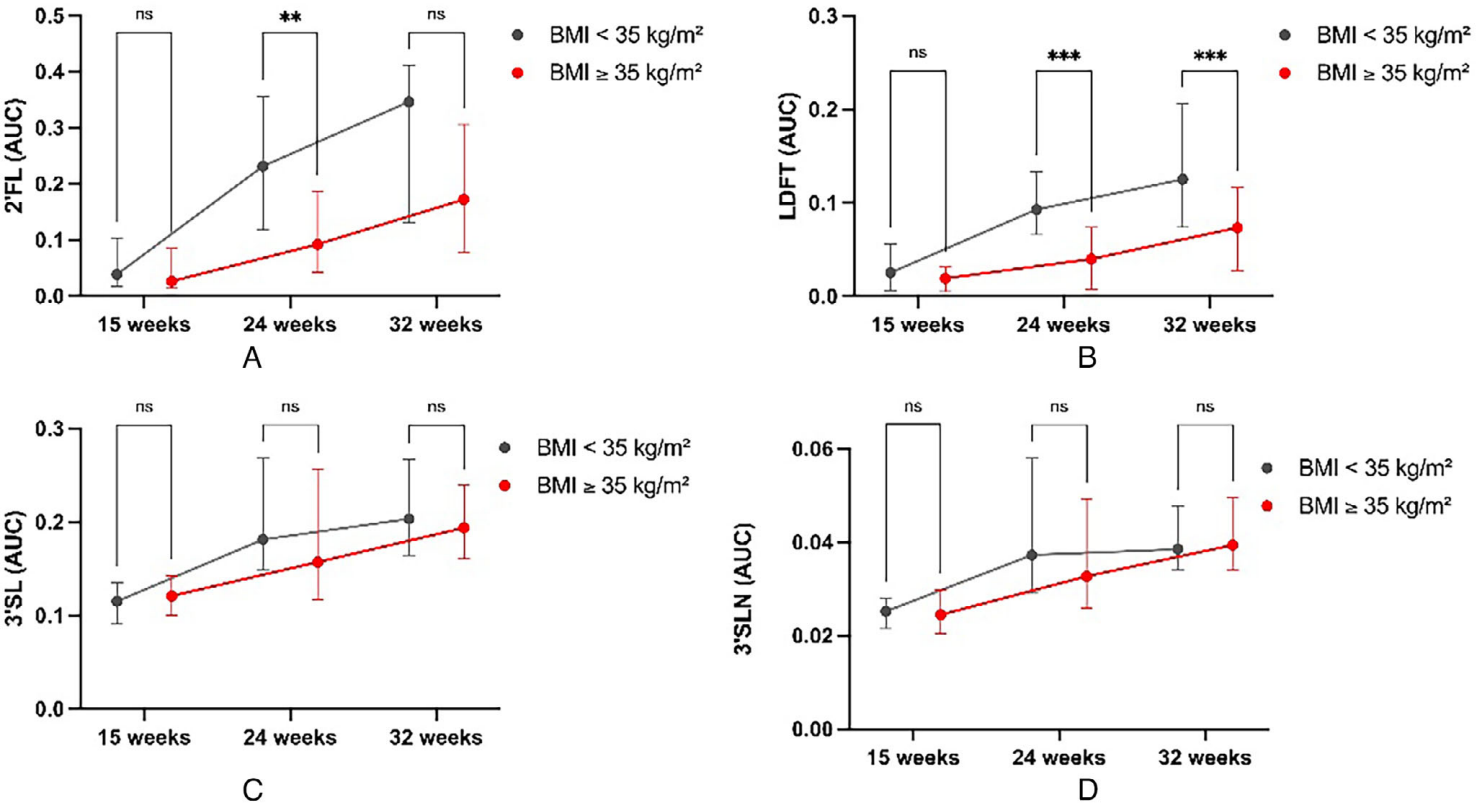
Trajectories of HMO concentrations in maternal serum (AUC normalized to internal standard) over the course of pregnancy. Line graphs show concentrations (median and IQR) separately for pregnant women with overweight/obesity class I (BMI < 35 kg m^−^^2^, gray lines) and with obesity class II and above (BMI ≥ 35 kg m^−^^2^, red lines) for the fucosylated HMOs 2’FL **(A)** and LDFT **(B)** (includes data of secretor‐positive women; BMI < 35 kg m^−^^2^, *n* = 39; BMI ≥ 35 kg m^−2^, *n* = 33) and the sialylated HMOs 3’SL **(C)** and 3’SLN **(D)** (data of the whole study sample, BMI < 35 kg m^−^^2^, *n* = 49; BMI ≥ 35 kg m^−2^, *n* = 38). Repeated measures 2‐way ANOVA (mixed model) with Sidak's multiple comparison test showed difference in concentration trajectories between the BMI categories for 2’FL and LDFT. Concentrations of 2’FL were significantly lower in women with obesity class II and above at week 24 (*p* = 0.0061). Concentrations of LDFT were significantly lower in women with obesity class II and above at week 24 and 32 (*p* < 0.0003). There were no significant differences in concentrations of 3’SL and 3’SLN at each time points between BMI groups. ns, not significant; **p < 0.001; ***p < 0.0001; HMO, human milk oligosaccharide; LDFT, Lactodifucotetraose; 2′FL, 2′‐Fucosyllactose; 3′SL, 3′‐Sialyllactose; 3′SLN, 3′‐Sialyllactosamine.

Stratifying the cohort into two BMI groups <35 kg m^−^^2^ (overweight and obesity class I) (*N* = 49) and ≥35 kg m^−^^2^ (obesity class II and above) (*N* = 38) showed no differences between HMO levels at 15 weeks. However, secretor‐positive women with obesity class II and above (BMI ≥ 35 kg m^−^^2^) showed significantly different HMO trajectories of fucosylated HMOs across pregnancy compared to secretor‐positive women with overweight/and class I obesity. Longitudinal changes in 2’FL and LDFT from 15 to 24 weeks were significantly reduced, resulting in lower median concentrations at 24 and 32 weeks of gestation (**Figure** **4**A,B). 3’SL and 3’SLN trajectories did not differ between BMI groups (Figure 4C,D).

Serum lipids at week 15 and 24 were not different between BMI groups, neither were HDL or triglycerides at week 32. However, total cholesterol and LDL were significantly lower in women with obesity class II and above (BMI ≥ 35 kg m^−^^2^) at week 32 (**Figure** **S1**, Supporting Information).

### 2.4 HMOs in Early Pregnancy Are Associated with Lipid Profiles Later in Pregnancy

We next investigated whether HMOs in early pregnancy were associated with the lipid profile. 2’FL at 15 weeks was positively associated with HDL concentrations at all three time points, also after adjustment for possible confounders (**Table** **4**). 2’FL was negatively associated with triglyceride concentrations at 24 and 32 weeks. 3’SL at 15 weeks was positively associated with total cholesterol and LDL concentrations at 15 and 24 weeks, and triglyceride concentrations at 15 weeks of gestation. LDFT was not associated with any of the lipid parameters at any time point. None of the HMOs were associated with changes in lipids from 15 to 24 or 32 weeks (data not shown).

**Table 4:** Associations of HMOs at 15 weeks with lipid parameters at three time points.

2’FL at week 15	Beta	95% CI	*p*	Beta	95% CI	*p*
15 weeks		Unadjusted *n* = 86			Adjusted *n* = 81	
Total cholesterol	0.77	−1.95, 3.49	0.58	0.87	−1.91, 3.66	0.53
LDL	−0.57	−3.06, 1.92	0.65	−0.36	−2.93, 2.20	0.78
HDL	**2.03**	**0.99, 3.15**	**0.001**	**1.92**	**0.76, 3.08**	**0.002**
Triglycerides	−1.52	−3.04, 0.001	0.05	−1.52	−1.34, 0.02	0.053
24 weeks		Unadjusted *n* = 85			Adjusted *n* = 77	
Total cholesterol	−0.68	−3.77, 2.42	0.67	−0.57	−3.71, 2.57	0.71
LDL	−1.39	−4.28, 1.49	0.34	−1.18	−4.17, 1.82	0.44
HDL	**1.71**	**0.41, 2.02**	**0.01**	**1.57**	**0.26, 2.88**	**0.02**
Triglycerides	**−2.21**	**−3.98, ‐0.45**	**0.01**	**−2.14**	**−3.91, −0.36**	**0.02**
32 weeks		Unadjusted *n* = 71			Adjusted *n* = 62	
Total cholesterol	−1.10	−4.48, 2.29	0.52	−1.12	−4.72, 2.51	0.54
LDL	−1.93	−5.14, 1.29	0.24	−1.42	−4.99, 2.14	0.43
HDL	**2.06**	**0.94, 3.19**	**0.001**	**1.72**	**0.51, 2.93**	**0.006**
Triglycerides	**−2.74**	**−4.87, −0.62**	**0.01**	**−3.12**	**−5.48, −0.76**	**0.01**
**LDFT at week 15**	**Beta**	**95% CI**	***p***	**Beta**	**95% CI**	***p***
15 weeks		Unadjusted *n* = 86			Adjusted *n* = 81	
Total cholesterol	−5.33	−12.95, 2.29	0.17	−6.93	−15.57, 1.71	0.11
LDL	−5.14	−12.11, 1.83	0.15	−7.60	−15.56, 0.37	0.06
HDL	−1.30	−4.46, 1.86	0.42	−0.87	−4.47, 2.72	0.63
Triglycerides	2.47	−1.78, 6.72	0.25	3.43	−1.34, 8.20	0.16
24 weeks		Unadjusted *n* = 85			Adjusted *n* = 77	
Total cholesterol	−2.32	−10.99, 6.36	0.60	−4.01	−13.73, 5.70	0.41
LDL	−2.25	−10.34, 5.84	0.58	−5.04	−14.31, 4.24	0.28
HDL	−1.36	−5.02, 2.30	0.46	−0.96	−5.02, 3.10	0.64
Triglycerides	2.88	−2.06, 7.82	0.25	4.41	−1.08, 9.89	0.11
32 weeks		Unadjusted *n* = 71			Adjusted *n* = 62	
Total cholesterol	−5.49	−16.45, 5.47	0.32	−7.40	−19.09, 4.30	0.21
LDL	−6.23	−16.62, 4.16	0.24	−7.78	−19.30, 3.75	0.18
HDL	−0.61	−4.26, 3.04	0.74	−1.01	−4.92, 2.90	0.61
Triglycerides	3.01	−3.85, 9.88	0.38	3.09	−4.55, 10.72	0.42
**3’SL at week 15**	**Beta**	**95% CI**	***p***	**Beta**	**95% CI**	***p***
15 weeks		Unadjusted *n* = 86			Adjusted *n* = 81	
Total cholesterol	**5.49**	**1.21, 9.76**	**0.01**	**−5.90**	**1.53, 10.28**	**0.01**
LDL	**4.21**	**0.30, 8.13**	**0.04**	**4.26**	**0.23, 8.29**	**0.04**
HDL	−0.55	−2.32, 1.22	0.54	−0.43	−2.25, 1.39	0.64
Triglycerides	**4.05**	**1.67, 6.43**	**0.001**	**4.60**	**2.18, 7.01**	**<0.001**
24 weeks		Unadjusted *n* = 85		Adjusted *n* = 77		
Total cholesterol	**5.04**	**0.20, 9.88**	**0.04**	**5.89**	**0.99, 5.70**	**0.02**
LDL	**4.59**	**0.07, 9.10**	**0.047**	**4.80**	**0.13, 9.48**	**0.044**
HDL	−0.45	−2.49, 1.59	0.66	0.03	−2.02, 2.08	0.98
Triglycerides	2.02	−0.74, 4.78	0.15	2.35	−0.42, 5.12	0.10
32 weeks		Unadjusted *n* = 71			Adjusted *n* = 62	
Total cholesterol	4.66	−0.75, 10.08	0.09	5.09	−0.48, 10.66	0.07
LDL	4.55	−0.58, 9.69	0.08	4.81	−0.68, 10.30	0.09
HDL	−0.33	−2.13, 1.48	0.72	−0.36	−2.22, 1.50	0.70
Triglycerides	0.97	−2.42, 4.37	0.57	1.43	−2.21, 5.07	0.43

Unadjusted models and adjusted models are presented. Adjusted models included maternal BMI, gestational weight gain, age, smoking, parity, and ethnicity. All three HMOs were entered into the models simultaneously. 3’SLN was excluded from the models because of high collinearity with 3’SL. Bold font indicates statistically significant associations.

### 2.5 Serum Lipids in Early Pregnancy Show Weak Association with HMOs Later in Pregnancy

To estimate the direction of the influence between serum lipids and HMOs in pregnancy, we also performed linear regression analysis with serum lipids as predictors for the HMOs at different time points. HDL levels at 15 weeks of gestation were positively associated with 2’FL concentrations at 32 weeks in an unadjusted model (**Table** **5**). Adjustment by BMI attenuated this association. No other associations of lipids at 15 weeks with HMOs were found; not for the whole sample and nor when only secretor positive women were included in the analyses.

**Table 5:** Associations of lipids at 15 weeks with HMOs at three time points.

LDL at week 15	Beta	95% CI	*p*	Beta	95% CI	*p*
15 weeks	Unadjusted *n* = 86				Adjusted *n* = 81	
2’FL	−0.10	−0.03, 0.01	0.33	−0.01	−0.03, 0.01	0.38
LDFT	**−0.01**	**−0.02, −0.000**	**0.043**	**−0.01**	**−0.02, −0.002**	**0.02**
3’SL	0.01	−0.002, 0.02	0.10	0.01	−0.001, 0.02	0.07
3’SLN	0.002	−0.001, 0.01	0.13	0.003	−0.001, 0.01	0.13
24 weeks	Unadjusted *n* = 84		Adjusted *n* = 79			
2’FL	−0.04	−0.09, 0.01	0.14	−0.05	−0.10, 0.003	0.07
LDFT	−0.01	−0.03, 0.01	0.30	−0.01	−0.03, 0.002	0.09
3’SL	0.01	−0.02, 0.03	0.57	−0.02	−0.22, 0.19	0.87
3’SLN	−0.001	−0.01, 0.01	0.79	0.001	−0.01, 0.01	0.72
32 weeks	Unadjusted *n* = 72		Adjusted *n* = 70			
2’FL	−0.05	−0.11, 0.01	0.10	−0.06	−0.12, 0.00	0.050
LDFT	−0.02	−0.04, 0.01	0.21	−0.02	−0.05, 0.003	0.08
3’SL	0.01	−0.01, 0.03	0.52	0.01	−0.02, 0.03	0.56
3’SLN	0.000	−0.01, 0.01	0.90	0.003	−0.003, 0.01	0.30
**HDL at week 15**	**Beta**	**95% CI**	***p***	**Beta**	**95% CI**	***p***
15 weeks		Unadjusted *n* = 86			Adjusted *n* = 81	
2’FL	**0.08**	**0.03, 0.13**	**0.001**	**0.08**	**0.03, 0.13**	**0.002**
LDFT	0.01	−0.01, 0.03	0.20	0.01	−0.01, 0.03	0.20
3’SL	0.01	−0.01, 0.04	0.28	0.02	−0.01, 0.04	0.22
3’SLN	0.01	−0.002, 0.01	0.14	0.01	−0.003, 0.01	0.19
24 weeks		Unadjusted *n* = 84	9		Adjusted *n* = 7	
2’FL	0.07	−0.04, 0.19	0.20	−0.15	−1.04, 0.73	0.73
LDFT	0.02	−0.02, 0.06	0.38	0.01	−0.03, 0.05	0.49
3’SL	−0.03	−0.08, 0.03	0.34	−0.03	−0.09, 0.03	0.34
3’SLN	−0.01	−0.02, 0.01	0.43	−0.01	−0.02, 0.01	0.50
32 weeks		Unadjusted *n* = 72			Adjusted *n* = 70	
2’FL	**0.13**	**0.002, 0.27**	**0.046**	0.14	−0.81, 1.09	0.77
LDFT	0.03	−0.03, 0.08	0.33	0.03	−0.03, 0.08	0.34
3’SL	−0.03	−0.07, 0.02	0.20	−0.03	−0.08, 0.02	0.29
3’SLN	−0.002	−0.027, 0.01	0.77	0.002	−0.01, 0.02	0.81
Triglycerides at week 15	Beta	95% CI	*p*	Beta	95% CI	*p*
15 weeks		Unadjusted *n* = 86			Adjusted *n* = 81	
2’FL	0.004	−0.03, 0.04	0.84	−0.003	−0.03, 0.04	0.89
LDFT	0.01	−0.01, 0.02	0.38	−0.43	−0.01, 0.02	0.26
3’SL	**0.03**	**0.01, 0.05**	**0.003**	**0.04**	**0.02, 0.06**	**0.001**
3’SLN	**0.01**	**0.001, 0.01**	**0.01**	**0.01**	**0.002, 0.01**	**0.01**
24 weeks		Unadjusted *n* = 84			Adjusted *n* = 79	
2’FL	0.03	−0.05, 0.12	0.47	0.03	−0.06, 0.12	0.47
LDFT	0.01	−0.02, 0.04	0.44	0.01	−0.02, 0.04	0.47
3’SL	−0.001	−0.04, 0.04	0.98	−0.001	−0.05, 0.05	0.96
3’SLN	0.000	−0.01, 0.01	0.94	0.000	−0.01, 0.01	0.97
32 weeks		Unadjusted *n* = 72			Adjusted *n* = 70	
2’FL	0.05	−0.05, 0.15	0.32	0.05	−0.05, 0.15	0.32
LDFT	0.02	−0.02, 0.06	0.41	0.02	−0.02, 0.06	0.36
3’SL	0.02	−0.02, 0.05	0.31	0.02	−0.02, 0.05	0.41
3’SLN	0.001	−0.01, 0.01	0.91	0.004	−0.01, 0.01	0.38

Unadjusted models and adjusted models are presented. Adjusted models included maternal BMI, gestational weight gain, age, smoking, parity, and ethnicity. All three lipids (HDL, LDL, triglycerides) were entered into the models simultaneously. Bold font indicates statistically significant associations.

## 3 Discussion

Our study focused on understanding the dynamic relationships between the most abundant prenatal HMOs and maternal pre‐pregnancy BMI and maternal metabolic health assessed as trajectories of body weight and circulating lipids. Taking into account the participants’ secretor status, we here report three key findings: 1) In women with a negative secretor status (lacking 2’FL and LDFT), weekly gestational weight gain was attenuated and BMI remained stable with pregnancy progression, in contrast to women with positive secretor status. 2) Women with secretor positive status and BMI of 35 kg m^−^^2^ or greater (obesity class II and above) showed a delay in the increase of fucosylated HMOs, 2’FL and LDFT, from early to mid‐pregnancy. 3) In the total study population, the sialylated HMO 3’SL in early pregnancy was associated with increased levels of total cholesterol and LDL in later pregnancy, while in women with secretor positive status, 2’FL in early pregnancy was associated with a more beneficial lipid profile in later gestation (higher HDL and lower triglycerides).

### 3.1 Pre‐Pregnancy BMI Influences HMO Trajectories, Maybe by Altered FUT2 Activity

Obesity and associated dyslipidemia are growing global health concerns associated with negative outcomes for mother and child.^[^ ^24^, ^25^ ^]^ In recent studies, higher maternal BMI was shown to be associated with an altered HMO profile in human milk^[^ ^33^ ^]^; and specific HMOs consumed with breast milk correlated with infant growth and adiposity,^[^ ^35^ ^]^ suggesting an impact of HMO composition on weight trajectories and lipid metabolism in breast fed infants. In the present study, we investigated the influence of pre‐pregnancy BMI on HMO concentrations during pregnancy. We found higher pre‐pregnancy BMI correlated with lower 2’FL and LDFT in women with overweight or obesity at mid‐pregnancy. This seems to fit well with a study in human milk, reporting on lower 2’FL concentrations in obese versus normal weight women.^[^ ^33^ ^]^ These results are also in line with our previous study in a small sample of healthy, normal weight pregnant women, finding negative associations of 2’FL at mid pregnancy with both maternal pre‐pregnancy BMI and subcutaneous adipose tissue.^[^ ^17^ ^]^

Stratifying the cohort into women with either overweight/obesity class I and women with obesity class II and above (BMI < 35 or ≥ 35 kg m^−^^2^) revealed significant differences in the trajectories of α‐1,2‐fucosylated HMOs (2’FL and LDFT) over the course of pregnancy. While concentrations in early pregnancy were not significantly different between the BMI groups, increases in 2’FL and LDFT with progressing gestation were smaller in women who had entered their pregnancies with obesity class II and above. This suggests that pre‐pregnancy obesity might influence the HMOs trajectories during pregnancy, potentially by modulating the secretor‐status dependent α‐1,2‐fucosylation. Biosynthesis of 2’FL and LDFT requires activity of FUT2, an enzyme encoded by the *Secretor* gene. Given a positive secretor status, a reduced production of these HMOs might be explained by either a limited availability of monosaccharide building blocks, decreased enzyme expression or reduced enzyme activity. Zhou et al. described a significant suppression of intestinal FUT2 in a mouse model of diet induced obesity,^[^ ^36^ ^]^ suggesting effects of maternal diet on the enzymatic activity of FUT2. Along this line, it is tempting to speculate that analogously, in secretor positive highly obese women, dietary habits might have downregulated the general activity of FUT2. In early pregnancy, a diet induced reduction in FUT2 activity might then also affect HMO production in the mammary gland, leading to a delayed increase of systemic 2’FL and LDFT.

### 3.2 Secretor Status Affects Gestational Weight Gain of Women with Overweight or Obesity

When stratifying our cohort in secretor positive and secretor negative women (based on 2’FL and LDFT concentrations that occur either in overall high amounts or are completely missing), we found no differences in pre‐pregnancy BMI. However, with progressing gestation, in secretor negative women the mean BMI showed hardly any change, whereas in secretor positive women the mean BMI significantly increased. Analogously, the weekly gestational weight gain between week 15 and 24 was significantly lower in secretor negative women, a trend that also continued between week 24 and 32 without reaching significance. This led to a significantly lower BMI at week 32 in secretor negative women compared to secretor positive counterparts. This suggests that in women with overweight or obesity, a negative secretor status might protect from additional weight gain, whereas a positive secretor status rather facilitates gestational weight gain. In line with this, the study by Zhou et al. showed that *Fut2* knockout mice (mimicking non‐secretor status) were resistant to diet induced obesity and liver steatosis, maybe due to an altered gut microbiome and different responses to metabolic challenges. The authors further proposed that functional downregulation of intestinal α1,2‐fucosylation upon Western diet might be a protective mechanism counteracting diet induced obesity and the metabolic syndrome. If this could be translated to humans, in secretor positive pregnant women with either overweight or obesity, a downregulation of FUT2 before pregnancy could be a protective mechanism to counterbalance diet induced metabolic derangements. Pregnancy might then abolish this protective effect of functional α1,2‐fucosylation downregulation in the intestine by increasing α1,2‐fucosylation in the mammary gland to produce pregnancy‐specific products of FUT2 (such as 2’FL or other α1,2‐fucosylated HMOs). Secretor negative women might remain protected against additional exaggerated gestational weight gain. However, it remains unclear whether it is fat mass that makes the difference in weight gain or other aspects of body composition. Thus, further studies investigating the role of FUT2 and its pregnancy related metabolites (such as 2’FL) in the context of weight gain, body composition, nutritional status, and metabolic adaptations during pregnancy need to be carried out to elucidate causal relationships.

### 3.3 2’FL May Influence Lipid Metabolism in Pregnancy Conferring an Atheroprotective Serum Lipid Profile

2’FL as one of the most abundant HMOs during gestation increases from the early second trimester on, accompanied by an increase in serum lipids and profound changes in the lipid metabolism. To assess the potential interaction of HMOs with serum lipids, we analyzed longitudinal associations of HMOs early in pregnancy with the lipid profile later. In our cohort of pregnant women with either overweight or obesity (characterized by both, pathologic and non‐pathologic levels of blood lipids), systemic 2’FL of week 15 was positively associated with HDL of all three time points and negatively with triglyceride concentrations at 24 and 32 weeks. Thus, 2’FL was associated with an atheroprotective lipid profile that is commonly believed to be beneficial for mother and fetus. 3’SL, on the contrary, was positively associated with total cholesterol and LDL concentrations at 15 and 24 weeks, and triglyceride concentrations at 15 weeks of gestation, representing the less advantageous lipid profile with elevated risks for cardiovascular diseases and pregnancy disorders.

Some previous animal studies have indicated an effect of 2’FL on lipid metabolism and weight gain. Gart et al. showed that 2’FL protected the liver against hepatic steatosis in obese mice by reducing microvesicular steatosis, partly mediated by activating the gene ACOX1 (involved in lipid catabolism) and deactivating SREBF1 (involved in lipogenesis).^[^ ^28^ ^]^ Similar results were found by Lee et al. in a mouse model of diet induced obesity. Oral supplementation of 2’FL led to decreased fat mass and body weight gain in high fat fed mice, and suppressed the upregulation of hepatic peroxisome proliferator‐activated receptor gamma, a transcription factor for adipogenesis.^[^ ^29^ ^]^ Although most of the effects on lipid metabolism were explained by altering the gut microbiome, direct effects of 2’FL on liver or other target cells seem also possible.

Taken together, the role of systemic 2’FL in terms of lipid metabolism and weight gain cannot be rated conclusively yet, but first studies indicate a potential involvement. To date, there are no other studies focusing on relationships between 2’FL or other HMOs and maternal serum lipid concentrations. Thus, we can of course only speculate about the role of circulating HMOs in pregnancy, the specific adaptations in lipid metabolism and potential causal relationships. Whether there are common influencing factors (e.g., nutrition, body weight) that affect both, HMO composition and lipid signature in pregnancy, or whether there is an active role of HMOs in the adaptation of the maternal lipid metabolism during pregnancy, remains to be further elucidated. Future studies focusing on anthropometrical and life‐style related influences on the HMO composition as well as investigating associations with other aspects of lipid or glucose metabolism will be interesting in this context. Addressing limitations of the present study it has to be noted that the original study per design only included women with overweight or obesity who are therefore characterized by an increased risk for metabolic disorders. Furthermore, due to a limited sample size, our analysis of secretor‐positive and ‐negative women needs to be seen as a pilot‐study, requiring confirmation in a larger, more heterogenous cohort of pregnant women (including normal weight participants). A larger cohort with a higher number of secretor negative women would help to further assess the impact of secretor status on the lipid trajectories throughout pregnancy. Finally, mechanistical experiments elucidating the role of systemic HMOs during pregnancy would be desirable. However, our descriptive data indicate a potential involvement of HMOs in metabolic adaptations during pregnancy. Our study also warrants further research addressing the interaction between maternal secretor status and susceptibility to metabolic disorders in pregnancy. In times of increasing health concerns related to obesity and dyslipidemia, these findings could be of future clinical importance.

## 4 Experimental Section

### Study Design

Study data and sample material had been collected during a randomized controlled trial investigating the effects of physical activity on insulin sensitivity and fasting glucose levels in a collective of pregnant women at high risk for gestational diabetes. The intervention group intended to carry out a special exercise regime twice a week, the control group did not.^[^ ^37^ ^]^ Since the primary outcome measures showed no effect of the intervention,^[^ ^38^ ^]^ the data of the well‐phenotyped cohort was further analyzed as a prospective longitudinal study. Recruitment of study participants and data collection was coordinated between three large hospitals and 10 midwifery practices in Amsterdam, The Netherlands, after approval by the Medical Ethics Committee of VU University Medical Centre in Amsterdam (2007/133).

### Study Participants

This was a secondary analysis of a study sample of pregnant women at increased risk for gestational diabetes (GDM). Inclusion criteria were obesity or overweight (pre‐pregnancy BMI ≥25 kg m^−^^2^) in combination with at least one of the following criteria: 1) history of macrosomia in previous pregnancies (birth weight >97th percentile of gestational age); 2) history of GDM; and 3) type 2 diabetes within first‐degree relatives.

Women were recruited between 14 and 20 weeks of gestation if they were over 18 years of age, fluent in the Dutch language, and willing to give written consent. Exclusion criteria were multiple pregnancy, hypertension, pre‐existing diabetes, and use of medication affecting insulin secretion or insulin sensitivity (e.g., corticosteroids, antivirals, antihypertensive drugs). Since the study aimed to generate and compare data throughout the whole pregnancy, only women recruited within the first 15 weeks of gestation were further included in the HMO analyses presented here.

### Procedures

Participants were examined at three time points throughout pregnancy (at 15, 24, and 32 weeks of gestation). The first visit included anamnesis (e.g., pre‐pregnancy body weight, smoking status, information about earlier pregnancies) and assessment of demographic variables (e.g., maternal age, ethnicity [Caucasian vs non‐Caucasian], parity, marital status, and educational level). Disclosure of anamnestic and/or personal information was not mandatory for the participants and therefore some data were missing. Physical measurements and laboratory tests were carried out at all three visits.

Calibrated electronic scales were used to measure the maternal body weight while women were wearing only indoor clothing without shoes. Barefooted body height was assessed by a wall‐mounted height scale. BMI was calculated as body weight in kilograms divided by the square of height in meters. For calculating the pre‐pregnancy BMI the self‐reported pre‐pregnancy weight was used. Gestational weight gain (GWG) was calculated as measured body weight at either 24 or 32 weeks minus measured body weight at 15 weeks. Weekly gestational weight gain was calculated as GWG divided by the exact corresponding difference between visits in days multiplied by seven. Excessive gestational weight gain was defined as a weight gain at 24 and 32 weeks that exceeded the IOM recommendations for pregnant women with overweight (0.3 kg week^−1^) or obesity (0.2 kg week^−1^).^[^ ^39^ ^]^

For laboratory tests fasting blood was drawn at all three time points from antebrachial or cubital veins after a fasting period of at least 10 h. Blood samples were centrifuged for 10 min at 1800 × *g*. Serum was either analyzed promptly (for lipid parameters) or stored at −80 °C (for HMOs).

### Lipid Parameters

Total cholesterol, triglycerides, and HDL were measured with commercial enzymatic kits on Roche/Hitachi modular P analyzers (Roche Diagnostics GmbH, Mannheim, Germany), whereas LDL was calculated using the Friedewald formula.^[^ ^40^ ^]^

### HMO Isolation from Blood Serum

Prenatal oligosaccharides were isolated from serum as previously described.^[^ ^18^ ^]^ Briefly, 50 μL of serum were diluted by 350 μL H_2_O containing Linear B6‐Trisaccharide (Dextra Laboratories) as an internal standard. The samples were subjected to two cycles of chloroform/methanol (2:1) extraction and subsequently to a solid phase extraction by using C18 columns (Thermo Fisher Scientific). The collected eluents were loaded onto graphitized carbon columns (Thermo Fisher Scientific), washed with H_2_O and eluted with 40% acetonitrile containing 0.05% trifluoroacetic acid.

### HMO Analysis by HPLC

Cleaned up and dried HMOs were labeled with the fluorescent tag 2‐aminobenzamid (2AB), as described earlier.^[^ ^41^ ^]^ The 2AB‐labeled HMOs were separated by HPLC on a TSKgel Amide‐80 column (Tosoh Bioscience, Japan) using a linear gradient of a 50 mM ammonium formate/acetonitrile buffer system. Fluorescently labeled glycans were detected by a fluorescence detector at 360 nm excitation and 425 nm emission. HPLC peaks were designated to HMOs by comparing retention times to those of commercially available standards of 2’‐Fucosyllactose (2’FL), Lactodifucotetraose (LDFT), 3’‐Sialyllactose (3’SL), and 3’‐Sialyllactosamine (3’SLN) (Prozyme, Hayward, CA, USA). Individual HMO concentrations were calculated by normalizing the area under the curve (AUC) of each HMO to the initially added internal standard (Linear B6‐Trisaccharide).

For further analyses, the study evaluated the secretor status of each woman by calculating the relative concentrations of 2’FL and LDFT. When both were below 10% of all HMOs, the woman was considered to be secretor negative.^[^ ^18^ ^]^

### Statistical Analyses

Data were presented as means and SD for normally distributed data and as median and IQR for skewed data. Spearman rank correlation coefficients were calculated to assess correlations between pre‐pregnancy BMI and HMO concentrations during pregnancy. Changes in HMOs over time were tested using Friedman ANOVA with Dunn's multiple comparison test and differences between BMI groups (<35 kg m^−^^2^; ≥35 kg m^−^^2^) with the Mann–Whitney *U*‐test. Secretor negative women were excluded from these analyses concerning 2’FL and LDFT, since they were lacking the capability of producing these HMOs.

Since the study aimed to identify possible causal relationships between HMOs and maternal lipids, it initially focused on associations of HMOs at 15 weeks with lipid outcomes at 15, 24, and 32 weeks of pregnancy. To assess the possible reversed direction of causality, the study subsequently assessed the associations of lipids at 15 weeks with HMO outcomes at 15, 24, and 32 weeks of pregnancy. Associations were assessed using linear regression models. Unadjusted models were presented and, in addition, fully adjusted models that included confounders known to influence lipid metabolism (maternal BMI, gestational weight gain, age, parity, smoking, and ethnicity). Very high collinearity between 3’SL and 3’SLN at 15 weeks was observed in most models, and the choice was made to include 3’SL in the models with lipid parameters as outcomes. All three HMOs (2’FL, LDFT, 3’SL), or all three types of lipids (HDL, LDL, triglycerides) were entered into the models simultaneously. Regression analyses were performed for the total study sample.

## Conflict of Interest

The authors declare no conflict of interest.

## Author Contributions

E.J.‐K. and M.v.P. contributed equally to this work. Conceptualization, M.v.P., L.S., E.J.‐K.; methodology, E.J.‐K., M.v.P., and H.K.; validation, E.J.‐K. and M.v.P.; formal analysis, L.S., E.J.‐K.; investigation, W.B., C.T., L.S., E.J.‐K.; resources, H.K., E.J.‐K., and M.v.P.; data curation, L.S., M.v.P., and E.J.‐K.; writing—original draft preparation, L.S., M.v.P.; writing—review and editing, H.F., M.v.P., and E.J.‐K.; visualization, L.S., E.J.‐K.; supervision, E.J.‐K.; funding acquisition, E.J.‐K. All authors have read and agreed to the published version of the manuscript.

## Supporting information

Supporting Information

## Data Availability

Data described in this article will be made available upon request. The data sets generated during and/or analyzed during the current study are not publicily available but are available from the corresponding author on reasonable request.
