# Supplementary material for: Prenatal Human Milk Oligosaccharides (HMOs) in the Context of BMI, Gestational Weight Gain, and Lipid Profile—An Association Study in Pregnant Women with Overweight or Obesity
Source: Mol Nutr Food Res. 2023 Dec 12;68(2):2300533. doi: 10.1002/mnfr.202300533 (PMC10909570; doi:10.1002/mnfr.202300533)
Supplement: Supplementary file 1 — Supporting Information [file MNFR-68-2300533-s001.pdf]

Supplementary Material

Associations of prenatal Human Milk Oligosaccharides (HMOs) with maternal BMI and lipid profile in overweight and obese pregnant women

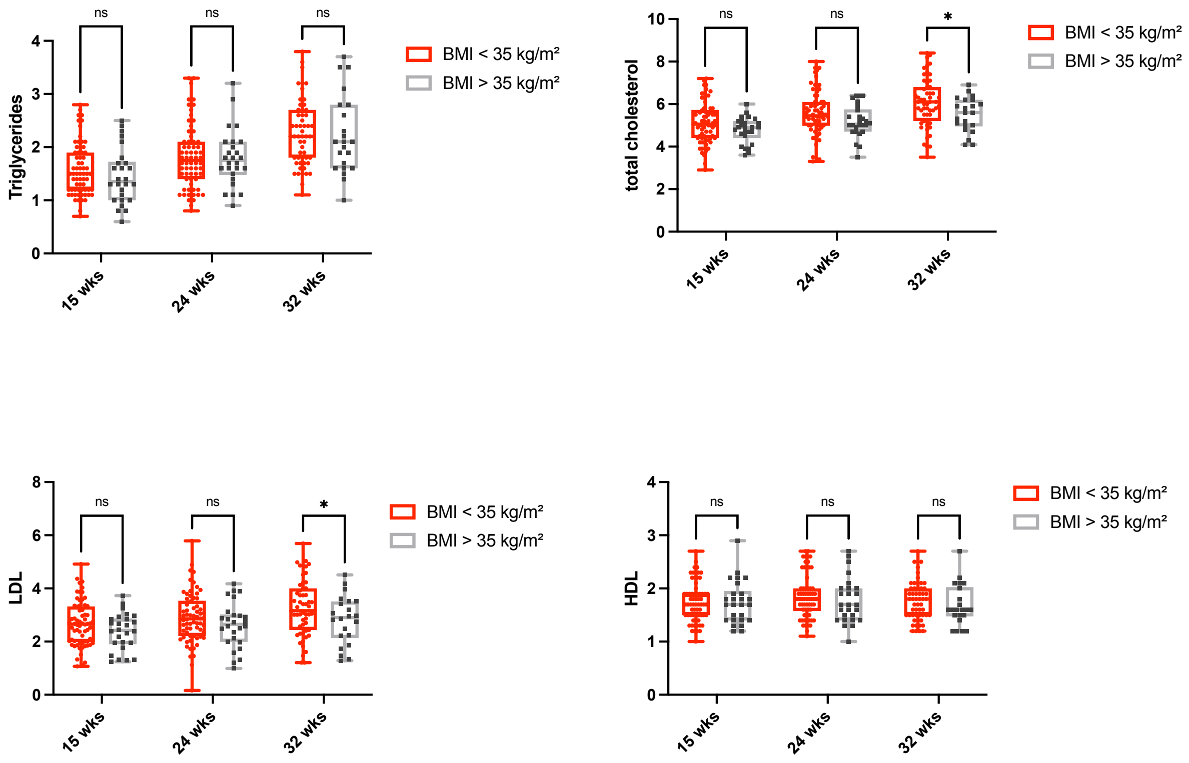

Supplementary Figure 1. Lipid concentrations over the course of pregnancy stratified by BMI < 35kg/m^2 and BMI ≥ 35kg/m^2. Box-and-whisker plots show concentrations in mmol/L for triglycerides, total cholesterol, LDL, and HDL separately for BMI groups at different time points during pregnancy. *p < 0.05; ns, not significant.
